# Motor Neuron Gene Therapy: Lessons from Spinal Muscular Atrophy for Amyotrophic Lateral Sclerosis

**DOI:** 10.3389/fnmol.2017.00405

**Published:** 2017-12-07

**Authors:** Andrew P. Tosolini, James N. Sleigh

**Affiliations:** Sobell Department of Motor Neuroscience and Movement Disorders, Institute of Neurology, University College London, London, United Kingdom

**Keywords:** adeno-associated virus (AAV), ALS, antisense oligonucleotide (ASO), motor neuron disease (MND), neurodegeneration, neurotrophic factor, SMA, survival motor neuron (SMN)

## Abstract

Spinal muscular atrophy (SMA) and amyotrophic lateral sclerosis (ALS) are severe nervous system diseases characterized by the degeneration of lower motor neurons. They share a number of additional pathological, cellular, and genetic parallels suggesting that mechanistic and clinical insights into one disorder may have value for the other. While there are currently no clinical ALS gene therapies, the splice-switching antisense oligonucleotide, nusinersen, was recently approved for SMA. This milestone was achieved through extensive pre-clinical research and patient trials, which together have spawned fundamental insights into motor neuron gene therapy. We have thus tried to distil key information garnered from SMA research, in the hope that it may stimulate a more directed approach to ALS gene therapy. Not only must the type of therapeutic (e.g., antisense oligonucleotide vs. viral vector) be sensibly selected, but considerable thought must be applied to the *where*, *which*, *what*, and *when* in order to enhance treatment benefit: to where (cell types and tissues) must the drug be delivered and how can this be best achieved? Which perturbed pathways must be corrected and can they be concurrently targeted? What dosing regime and concentration should be used? When should medication be administered? These questions are intuitive, but central to identifying and optimizing a successful gene therapy. Providing definitive solutions to these quandaries will be difficult, but clear thinking about therapeutic testing is necessary if we are to have the best chance of developing viable ALS gene therapies and improving upon early generation SMA treatments.

## Introduction

Spinal muscular atrophy and amyotrophic lateral sclerosis are two devastating neurological conditions with the common pathological hallmark of motor neuron degeneration, ultimately leading to muscle wasting and death. While etiology, age of onset, progression, and survival outcomes can drastically differ between the diseases, they share a number mechanistic parallels; thus, experimental and clinical insights into the one disorder may prove useful for the other.

It is an exciting time for the SMA community as the first treatment, an ASO gene therapy called nusinersen, was approved in the US by the FDA on 23rd December, 2016. Nusinersen subsequently received marketing authorisation in the EU from the EMA in June, 2017. This starkly contrasts with the situation for ALS, where clinically viable gene therapies are currently non-existent, while recent trials of chemically diverse drugs have failed to live up to expectations piqued by mouse experiments. Although there are numerous complications in treating ALS that do not pertain to SMA, a number of fundamental lessons have been learnt from the gamut of pre-clinical research and clinical trials of SMA gene therapies that could prove useful in galvanizing a targeted approach to ALS gene therapy design and development.

In this review, we will first provide introductions to SMA, ALS, and commonalities between the two, and follow this with an overview of gene therapies tested in clinical trials for both diseases. We use the term gene therapy to encompass both virus-mediated gene transfer and ASO gene targeting. Rather than provide an exhaustive review of all SMA and ALS gene therapy research, which has been collectively well covered (Federici and Boulis, 2012; Nizzardo et al., 2012; Mulcahy et al., 2014; Scarrott et al., 2015; Singh N.N. et al., 2017), we will then outline some of the major issues that SMA gene therapy has encountered, try to distil key, emergent concepts, and frame this in the context of ALS in order to provide possible future experimental directions.

## Diseases of Motor Neurons: Genetics, Classifications, And Mechanisms

### Spinal Muscular Atrophy

**FIGURE 1:**
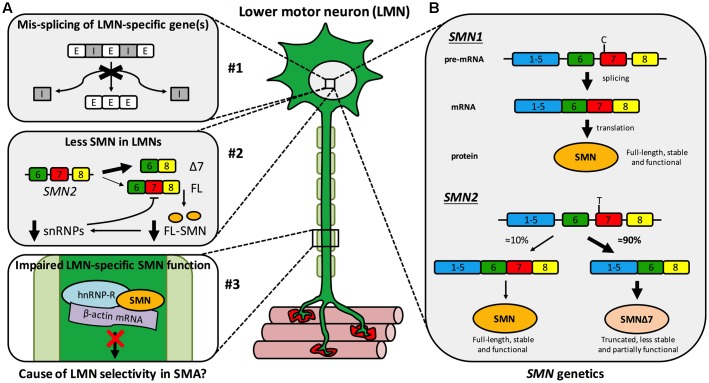
Spinal muscular atrophy mechanisms and genetics. **(A)** Multiple hypothetical mechanisms have been suggested to cause or contribute to the selective LMN degeneration of SMA, the three most plausible of which are that: #1 SMN reduction impairs splicing fidelity of a LMN-specific gene(s). E, exon; I, intron; #2 less SMN is available in LMNs than other cells due to a negative feedback loop, more prominent in LMNs, in which low SMN levels impair further *SMN2* exon 7 inclusion (see **B**) exacerbating SMN reduction. Δ7, truncated *SMN* mRNA; FL, full-length *SMN* mRNA; FL-SMN, full-length SMN protein; snRNPs, small nuclear ribonucleoproteins; and #3 a non-canonical, LMN-specific function of SMN is perturbed, for instance anterograde axonal transport of mRNA. **(B)** A single copy of *SMN1* produces enough SMN protein for motor neurons to thrive and for SMA disease carriers to remain healthy. Due to a single base pair distinction (C-to-T transition in exon 7), exon 7 is mis-spliced out of ≈90% of *SMN2* transcripts, resulting in a truncated, less stable, and only partially functional protein. This figure has been adapted from *Neuromuscular Disorders*, 23, Sleigh et al., 2013 Spinal muscular atrophy at the crossroads of basic science and therapy, 96, Copyright (2013), with permission from Elsevier.

Spinal muscular atrophy (SMA) is a monogenic neuromuscular disorder affecting ≈1 in 8,500–12,500 newborns, and is the most common genetic cause of infant mortality (Verhaart et al., 2017a,b). Patients present with severe muscle weakness and atrophy, predominantly in proximal (e.g., trunk) muscles, due to degeneration of LMNs of the spinal cord ventral horn. Pathology in additional cells and tissues can be observed in more severe manifestations of the disease, which has considerable implications for treatment (Hamilton and Gillingwater, 2013). SMA is caused by reduced levels of SMN protein (Lefebvre et al., 1995), which is found in the nucleus and cytoplasm of almost all cells, and plays a vital, canonical, housekeeping role in spliceosome assembly (Fischer et al., 1997; Liu et al., 1997; Pellizzoni et al., 2002), amongst other functions (Singh R.N. et al., 2017). Specifically, as part of the multi-protein SMN complex, SMN directs the efficient cytoplasmic assemblage of small nuclear RNAs (snRNAs) with Sm protein rings leading to the formation of small nuclear ribonucleoproteins (snRNPs) (Gruss et al., 2017). After nuclear-import, snRNPs function in the catalytic removal of introns from pre-mRNA transcripts in the process of splicing (Pellizzoni et al., 2002). Despite having a good understanding of this and other functions of SMN, the precise cause of the largely selective LMN death remains to be fully resolved. It is likely that a combination of multiple mechanisms account for this vulnerability, including mis-splicing of LMN-specific genes, SMN levels being lower in LMNs than other cell types, and disturbance of a possible non-canonical, LMN-specific SMN function (**Figure 1A**) (Tisdale and Pellizzoni, 2015; Jablonka and Sendtner, 2017; Tu et al., 2017).

Survival motor neuron is encoded by two almost identical genes, *SMN1* and its paralogue *SMN2* (**Figure 1B**) (Lefebvre et al., 1995). A single functioning *SMN1* allele produces sufficient protein for LMNs to remain healthy – as demonstrated by the ≈1 in 40–60 people with one functional copy of *SMN1* (i.e., SMA carriers) showing no clinical phenotype. However, due to a single nucleotide distinction (synonymous C-to-T alteration 6 nucleotides into exon 7), exon 7 of *SMN2* is aberrantly spliced ≈90% of the time (creating truncated, non-functional SMNΔ7 protein) and is therefore capable of producing only ≈10% of the full-length SMN made by *SMN1* (**Figure 1B**) (Lorson et al., 1999; Monani et al., 1999). Thus, when protein production from *SMN1* is impaired, as it is in SMA patients, *SMN2* can only partially compensate. SMN is highly conserved throughout evolution, permitting modeling of reduced SMN function in diverse organisms (Grice et al., 2011; Patten et al., 2014; Duque et al., 2015); however, all models naturally only possess an ortholog of *SMN1*, but not *SMN2*. To better mimic disease genetics, many transgenic mice have been engineered to express diminished SMN levels (Sleigh et al., 2011), the most frequently used being the SMNΔ7 mouse, which combines human *SMN2* and *SMN*Δ*7* transgenes on a null *Smn* background (Le et al., 2005). Relative genomic instability of the region (5q13) is believed to be the reason for the recent evolutionary duplication of the *SMN* locus (Rochette et al., 2001), and may also account for there being considerable variability in *SMN2* copy number within the population. The number of *SMN2* genes a patient with SMA possesses has important ramifications for disease severity, as more *SMN2* copies can produce more SMN, which correlates with diminished symptom severity (Lefebvre et al., 1997). Although not predictive at the individual level, in a population, *SMN2* copy number thus inversely correlates with SMA severity (McAndrew et al., 1997), which is categorised into four principal post-natal types (I-IV) based on age of onset and motor milestones achieved (Munsat and Davies, 1992).

Manifesting at or before 6 months and radically limiting life expectancy (<2 years), type I SMA (a.k.a. Werdnig-Hoffmann disease) is the most severe and frequently diagnosed form of SMA, and prevents children from ever being able to sit unaided. Infant death is usually caused by respiratory complications, although with specialized care, lifespan can be artificially extended for long periods. Type II SMA (Intermediate/Dubowitz Syndrome) presents between 7 and 18 months, permits unaided sitting but not walking, and has survival probabilities of ≈93% and ≈52% at 20 and 40 years, respectively (Farrar et al., 2013). Type III SMA (Kugelberg-Welander disease) limits motor function and has an onset > 18 months, but before adolescence, while type IV SMA (adult-onset) typically manifests in the second or third decade of life with mild-to-moderate muscle weakness, but generally no respiratory issues.

### Amyotrophic Lateral Sclerosis

With a lifetime risk of ≈1 in 400 (Alonso et al., 2009), ALS, also called MND and Lou Gehrig’s disease, is a fatal, progressive, mostly adult-onset disorder of both LMNs and UMNs. Neurodegeneration is observed in the cortex, corticospinal tracts, brainstem, and spinal ventral horn neurons and is accompanied by neuroinflammation (Brown and Al-Chalabi, 2017). Starting focally and spreading, this causes major symptoms of muscle weakness and fasciculations with subsequent atrophy, leading to death usually through respiratory failure within 3 years of diagnosis (Chiò et al., 2009). ALS also shares neuropathological and genetic features with FTD (Morita et al., 2006; Neumann et al., 2006; Vance et al., 2006), with approximately half of ALS patients showing some level of cognitive impairment (Ringholz et al., 2005). This has led to ALS and FTD being considered as part of the same clinicopathological spectrum (Ling et al., 2013). ALS patients display considerable symptom heterogeneity inclusive of age, site of disease onset, the rate and pattern of spread, and relative LMN/UMN involvement (Kiernan et al., 2011). ALS can therefore be sub-categorized based on several clinical and neuropathological criteria (Al-Chalabi and Hardiman, 2013; Bäumer et al., 2014). Given this variability, the ALS diagnosis is challenging, particularly during early disease stages (Byrne et al., 2012). The mean time from first noticeable symptom to clinical diagnosis is consequently (≈1 year). Contributing to this delay, no diagnostic or prognostic biomarkers are yet in regular clinical use for ALS, which also impacts the assessment of therapeutic efficacy in patient trials (Rosenfeld and Strong, 2015). Nevertheless, a number of prospective biomarkers have recently been identified, including the neurotrophin receptor p75^NTR^ extracellular domain in urine (Shepheard et al., 2017) and neurofilament chains in plasma (Lu et al., 2015) and CSF (Oeckl et al., 2016).

ALS has traditionally been classified into clinically indistinguishable sporadic (sALS) and familial (fALS) forms; sALS occurs without family history of the disease and represents the majority of cases (≈90%), whereas fALS contributes ≈10% of patients and is genetically inherited, predominantly in an autosomal, dominant fashion. The pathological and clinical variability of the disease has led to the idea that, in addition to being on a continuum with FTD, ALS itself may not be a single disorder, but a syndrome (Turner et al., 2013). Consistent with this, aberrations in over 25 genetic loci have been reproducibly linked with the ALS phenotype (Brown and Al-Chalabi, 2017), with new genes constantly being identified (Freischmidt et al., 2015; Brenner et al., 2016; Mackenzie et al., 2017). The four most common mutations are large, intronic, hexanucleotide repeat (G_4_C_2_) expansions in *chromosome 9 open reading frame 72* (*C9orf72*) (DeJesus-Hernandez et al., 2011; Renton et al., 2011), and dominant mutations in *superoxide dismutase 1* (*SOD1*) (Rosen et al., 1993), *transactive-region DNA binding protein* (*TARDBP* encoding TDP-43) (Sreedharan et al., 2008), and *fused in sarcoma* (*FUS*, a.k.a. *translocated in liposarcoma*, *TLS*) (Kwiatkowski et al., 2009; Vance et al., 2009). Mutations in *C9orf72* are the most common genetic cause of ALS hitherto identified, accounting for ≈40% of fALS and ≈7% of sALS (in populations of European ancestry) (Renton et al., 2014). The exact function of the encoded protein remains unclear, but it appears that it may be important in membrane trafficking and autophagy (Nassif et al., 2017). Encoding a Cu/Zn dismutase enzyme that provides defense against toxic superoxide free radicals, *SOD1* was the first gene linked to ALS (Rosen et al., 1993), and its mutation is responsible for ≈12% and ≈1% of fALS and sALS patients, respectively (Renton et al., 2014). As a consequence of its early identification and the rapid generation of the SOD1^G93A^ mouse model (Gurney et al., 1994), research into *SOD1* has shaped much of the ALS research landscape. Nevertheless, many other cellular and animal models are now available for different genetic forms of the disease (van Damme et al., 2017). *TARDBP* and *FUS* encode nucleic acid-binding proteins that predominantly reside in the nucleus, and are involved in multiple aspects of RNA processing, such as transcription and splicing. Mutations in these two genes each account for ≈4% of fALS and ≈1% of sALS patients (Renton et al., 2014). Despite significant progress in our understanding of the molecular pathogenesis linked to these four genes, it has not been fully resolved as to whether pathology is solely caused by a toxic gain-of-function or whether there are also loss-of-function effects (Lee et al., 2012; Bunton-Stasyshyn et al., 2015; Scekic-Zahirovic et al., 2016; Moens et al., 2017).

Causative genetic mutations have been identified in only ≈68% and ≈11% of fALS and sALS patients, respectively (Renton et al., 2014). This lack of an obvious genetic cause in most ALS patients, along with incomplete penetrance in several fALS pedigrees (Cirulli et al., 2015; Freischmidt et al., 2015), suggests that ALS may most frequently arise from additive effects of an assortment of predispositions and insults (Al-Chalabi and Hardiman, 2013). Indeed, rare variants in many other genes have been identified as ALS risk factors (van Rheenen et al., 2016), as have particular environmental stimuli (Martin et al., 2017a). Moreover, twin studies indicate that sALS heritability in the absence of a family history of the disease is still ≈60% (Al-Chalabi et al., 2010). Together, these data indicate that ALS may develop through a multi-step process in which aging is a critical component (Al-Chalabi et al., 2014), involving varying degrees of heritability and diverse, but inter-related, functional pathways that, upon dysregulation, yield motor neuron degeneration (Turner et al., 2013). This makes the strict fALS/sALS distinction an artificial dichotomy (Talbot, 2011), and presents obvious and considerable hurdles for the identification and development of viable therapeutic strategies for the disease.

Intrinsic motor neuron defects and non-cell autonomous toxicities in associated cell types (e.g., glia, interneurons) contribute to ALS (Ilieva et al., 2009; Ramírez-Jarquín et al., 2014; Puentes et al., 2016), but similar to SMA, the exact mechanisms underpinning motor neuron death, and their relative vulnerability-resistance axis (Nijssen et al., 2017), remain to be elucidated. Nevertheless, given the known functions of major ALS genes, altered RNA processing, nuclear protein mishandling/protein quality control, and impaired cytoskeletal dynamics appear to be three inter-related central themes (Bäumer et al., 2010; Brown and Al-Chalabi, 2017). Congruously, the vast majority of both sALS and fALS patients display cytoplasmic depositions of aggregated proteins, the main component of which is TDP-43 (Neumann et al., 2006), albeit with varied cellular distributions (Al-Sarraj et al., 2011). However, these inclusions conspicuously lack TDP-43 in *SOD1*-linked (Mackenzie et al., 2007) and *FUS*-linked (Vance et al., 2009) ALS. Additional defects in diverse cellular processes have been implicated in ALS including excitotoxicity, oxidative stress, altered oligodendrocyte function, axonal transport defects, mitochondrial malfunction, and neurotrophic factor deficits (reviewed in Kiernan et al., 2011; Taylor et al., 2016). It remains unclear as to which, if any, of these phenomena play a primary role in disease pathogenesis, rather than simply being non-specific consequences of a dysfunctional system. Moreover, it should be noted that most of these pathologies were identified using SOD1^G93A^ mice, which have their limitations for modeling all forms of ALS (Kiernan et al., 2011; Turner et al., 2013). To overcome this, numerous transgenic mouse models of ALS have been developed (van Damme et al., 2017), and strict guidelines for their use in pre-clinical therapeutic trials have been created to limit irreproducibility (Ludolph et al., 2010).

### SMA and ALS: A Common Mechanism?

SMA and ALS share a propensity for LMN degeneration leading to muscle wasting and atrophy. A number of key cellular and molecular parallels between the two diseases have also been reported (Cauchi, 2014; Gama-Carvalho et al., 2017; Hensel and Claus, 2017). The causative gene in SMA encodes a widely expressed, multi-functional protein important for fundamental cellular processes including pre-mRNA splicing (Fischer et al., 1997; Liu et al., 1997; Pellizzoni et al., 2002), transcription (Pellizzoni et al., 2001), and mRNA transport and stability (Rossoll et al., 2003; Zhang et al., 2003). While the importance of protein quality control to ALS should not be underestimated, some of the major genetic contributors to the disease, perhaps with the exception of C9ORF72, are also found ubiquitously and perform similar functions vital to RNA processing and maturation (Bäumer et al., 2010). For instance, both TDP-43 and FUS are involved in splicing (Zhou et al., 2002; Polymenidou et al., 2011) and transcription (Uranishi et al., 2001), and, along with SOD1, are thought to be important for the transport and stability of mRNA (Fujii and Takumi, 2005; Lu et al., 2007; Strong et al., 2007). Recently, dominant mutations in *T-cell restricted intracellular antigen 1* (*TIA1*), which is an RNA-binding protein involved in *SMN2* exon 7 splicing (Singh et al., 2011), were shown to play a causative role in ALS (Mackenzie et al., 2017), while *TIA1* knockout modifies phenotypes of mild male SMA mice (Howell et al., 2017). Furthermore, wild-type TDP-43 and FUS interact with SMN (Wang I.-F. et al., 2002; Yamazaki et al., 2012; Groen et al., 2013; Sun et al., 2015), and all three proteins have been implicated in the formation of stress granules (Hua and Zhou, 2004; Andersson et al., 2008; Colombrita et al., 2009), as has C9ORF72 (Maharjan et al., 2017).

Comparable in structure to stress granules, Gemini of Cajal bodies (a.k.a. gems) are membrane-free, nuclear conglomerates of SMN and associated proteins (Liu and Dreyfuss, 1996), the number of which correlates with SMN availability and is thus inversely related to SMA severity (Lefebvre et al., 1997; Feng et al., 2005). Numerous studies have shown that gem distribution/number is also affected in SOD1-, FUS-, and TDP-43-associated ALS patient tissue, mice, and cellular models (Shan et al., 2010; Gertz et al., 2012; Kariya et al., 2012; Yamazaki et al., 2012; Ishihara et al., 2013; Tsuiji et al., 2013; Sun et al., 2015), although this pattern was not observed in sALS patient fibroblasts (Kariya et al., 2014b). Nonetheless, SMN protein levels are reduced in sALS patient spinal cords (Turner et al., 2014) and pre-symptomatically in SOD1^G93A^ mouse spinal cords, while a 50% reduction in SMN exacerbates the SOD1^G93A^ phenotype (Turner et al., 2009). Furthermore, one study showed that ALS patients on average possess fewer *SMN2* copies, while having one *SMN1* copy is associated with increased ALS susceptibility (Veldink et al., 2005), although this link is not straightforward (Blauw et al., 2012; Corcia et al., 2012; Wang X.-B. et al., 2014). Nevertheless, SMN upregulation protects against mutant SOD1-induced cell death in an immortalized motor neuronal cell line (NSC-34) (Zou et al., 2007), rescues mutant FUS-mediated axonal defects in primary cortical neurons (Groen et al., 2013), and can improve survival of iPSC-derived motor neurons differentiated from SOD1 and TDP-43 ALS patient fibroblasts (Rodriguez-Muela et al., 2017). Moreover, neuronal SMN overexpression aids motor neuron survival and delays symptom onset in SOD1^G85A^, SOD1^G93A^, and TDP-43^A315T^ mice (Kariya et al., 2012; Turner et al., 2014; Perera et al., 2016). Survival of both SOD1 models was unaffected, but female TDP-43 mice displayed a significant extension. SMN may in fact serve as a general survival factor for motor neurons, as it is required to facilitate neuromuscular regeneration post-sciatic nerve crush in adult mice (Kariya et al., 2014a). In addition, low SMN levels in healthy iPSC-derived motor neurons correlate with greater cell death, and SMN upregulation promotes increased survival of control motor, but not cortical, neurons (Rodriguez-Muela et al., 2017).

These commonalities between SMA and ALS suggest that a shared mechanism could underlie at least certain aspects of the two diseases. Perhaps the most likely cause of the link is sequestration of SMN and/or splicing factors into cytoplasmic inclusions by mutant ALS gene products, resulting in defective RNA homeostasis. Indeed, ALS-associated mutations in FUS can enhance its association with SMN and impinge upon its axonal localization (Groen et al., 2013; Sun et al., 2015). Additionally, mutant FUS and *C9orf72* expansion can affect splicing factor distribution (Gerbino et al., 2013; Lee et al., 2013; Mori et al., 2013; Yu et al., 2015; Reber et al., 2016), as can homozygous overexpression of human wild-type FUS in mice (Mirra et al., 2017). Widespread splicing defects are unlikely to account for the motor neuron selectivity observed in SMA (Bäumer et al., 2009); however, the early and specific mis-splicing of a few crucial motor neuron-expressed genes may be particularly relevant to disease pathogenesis (Zhang et al., 2013; Sleigh et al., 2014). Splicing impairments have also been reported in ALS models and patient tissue (Chabot and Shkreta, 2016; Conlon et al., 2016); it is thus plausible that early splicing perturbations in a common set of critical genes could explain some of the shared pathomechanisms of SMA and ALS. Indeed, considerable overlap in alternative splicing events between SMA models and human FUS-expressing mice were recently reported, including in a number of ALS-pertinent genes (Mirra et al., 2017). These discoveries and the mechanistic intersection of ALS with SMA, suggest that gene therapy strategies able to augment SMN levels may be beneficial to both fALS and sALS patients, perhaps not in isolation, but as part of a combinatorial approach.

## Clinical Gene Therapy for Motor Neuron Diseases

### SMA: SMN Restoration Is Key

The genetic lesion underlying SMA causes diminished SMN protein levels; in theory, treatment is thus simple – replenish SMN. Small molecule, *SMN2* splice-modifying drugs, such as RG7916 (Roche) and LMI070 (Novartis), that augment SMN and SMN-independent, neuroprotection strategies are being pursued (Scoto et al., 2017), but *SMN* gene therapies are currently proving more clinically promising. In the last decade, we have rapidly transitioned from several early ineffective SMA patient trials (Fuller et al., 2010), to the recent regulatory approval of nusinersen for the treatment of SMA types I-IV.

**FIGURE 2:**
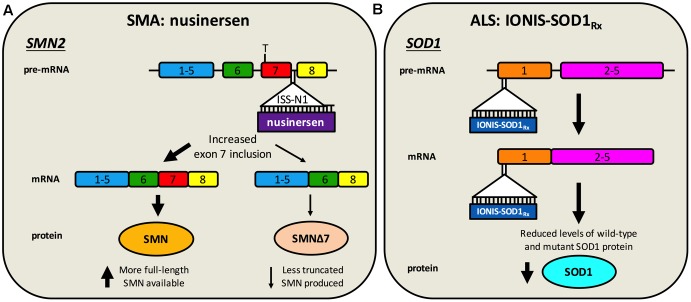
Antisense oligonucleotide targeting of *SMN2* splicing and *SOD1* translation. **(A)** Nusinersen is a SSO complementary to an intronic sequence in *SMN2* called ISS-N1, which is the main inhibitory element for exon 7 splicing. Hybridisation of nusinersen to ISS-N1 within pre-mRNA causes more frequent inclusion of exon 7 in mature *SMN2* transcripts, leading to an increase in the production of full-length SMN protein. Nusinersen is FDA- and EMA- approved for the treatment of SMA. **(B)** IONIS-SOD1_Rx_ specifically targets a 20 nucleotide-long sequence within exon 1 of *SOD1*, resulting in binding to both pre-mRNA and mature RNA causing inhibition of wild-type and mutant *SOD1* expression. ALS patients are currently being recruited for a phase Ib/IIa trial of IONIS-SOD1_Rx_.

Nusinersen (a.k.a. Spinraza, IONIS-SMN_Rx_, ISIS-SMN_Rx_, ISIS 396443, and ASO-10-27) is an ASO developed through work of numerous laboratories and a collaboration between Biogen Idec and Ionis Pharmaceuticals (formerly Isis Pharmaceuticals). ASOs are short (15–25 nucleotides), synthetic, single-stranded DNA or RNA sequences that specifically bind to target pre-mRNA or mRNA sequences, impacting gene expression. ASOs that specifically modulate splicing are also called SSOs. Importantly for diseases affecting the nervous system, ASOs distribute widely when injected into the CSF, do not require carrier molecules, and have relatively long half-lives (Geary et al., 2015). Nusinersen, which is delivered via single intrathecal injections directly into the CSF (i.e., by lumbar puncture) (Chiriboga et al., 2016; Haché et al., 2016), is a 2′-*O*-(2-methoxyethyl) modified ASO complementary to the ISS-N1 found in intron 7 of *SMN2* pre-mRNA (Singh et al., 2006; Hua et al., 2008). Specific ASO/pre-mRNA hybridisation restricts exon 7 mis-splicing, thereby increasing the amount of functional SMN made by *SMN2* (**Figure 2A**). A considerable amount of work in SMA mice provided substantial evidence for *in vivo* efficacy of nusinersen (Singh N.N. et al., 2017), leading to a series of stratified clinical trials (Chiriboga et al., 2016; Finkel et al., 2016, 2017). A pre-specified interim analysis from a randomized, double-blind, sham procedure-controlled phase III trial in SMA type I patients called ENDEAR (ClinicalTrials.gov Identifier: NCT02193074), provided enough evidence in mid-2016 that nusinersen caused statistically significant improvements in motor function (Finkel et al., 2017), prompting submission to the FDA of the new drug application. Interim analyses from a second phase III trial with type II SMA patients called CHERISH (NCT02292537) and an open-label study in pre-symptomatic infants named NURTURE (NCT02386553) have also proven encouraging.

A second gene therapy called AVXS-101 (a.k.a. scAAV9.CB.SMN and ChariSMA), which delivers the *SMN1* gene using non-replicating self-complementary adeno-associated virus serotype 9 (scAAV9), has also shown significant pre-clinical potential (Mulcahy et al., 2014). A major advantage of this therapy over nusinersen is that AAV9 can cross the BBB in mice, cats, and non-human primates, permitting intravenous delivery (Duque et al., 2009; Foust et al., 2009; Samaranch et al., 2012). Moreover, AAV9 displays neuronal tropism and can mediate stable, long-term expression with a single administration, which is important given immunogenicity issues associated with viruses (Lorain et al., 2008). This contrasts with the multiple, invasive intrathecal injections of nusinersen, which can have adverse side effects (Haché et al., 2016). Marketed by AveXis, AVXS-101 has completed testing in type I SMA patients in an open-label, dose-escalation phase I clinical trial (NCT02122952). The treatment is safe and well tolerated, and caused improvements in survival, attainment of motor milestones, and motor function when compared with historical SMA type I cohorts (Mendell et al., 2017). Two further open-label phase III trials with type I patients in the US and EU are planned. *SMN1* expression is driven by a hybrid cytomegalovirus enhancer/chicken β-actin (CAG) promoter, and AVXS-101 is being injected intravenously.

### ALS: A Gene Therapy on the Horizon?

No drugs are currently being tested in late-stage clinical trials for ALS, and until recently, the only FDA-/EMA-approved drug for the disease was the orally available Riluzole, which prolongs patient survival by ≈3 months (Bellingham, 2011). Riluzole can influence ion channel function, neurotransmission, and growth factor secretion, but inhibition of glutamate release from pre-synaptic nerve terminals counteracting motor neuron excitotoxicity appears to be the most disease-relevant benefit (Bellingham, 2011). In May 2017, the FDA surprisingly approved Edaravone (a.k.a. MCI-186 and Radicava), which is a free radical scavenger shown to modestly slow symptom progression in SOD1^G93A^ mice (Ito et al., 2008). In an open-label phase II study without comparator arm involving 19 ALS patients, intravenous administration of Edaravone was shown to be safe and reduce oxidative stress (Yoshino and Kimura, 2006); however, in a subsequent double-blind, placebo-controlled study, drug efficacy was not demonstrated (Abe et al., 2014). Nevertheless, a phase III trial with narrow inclusion criteria showed Edaravone modestly delayed disease progression in a limited subset of ALS patients (Writing Group and Edaravone (MCI-186) ALS 19 Study Group, 2017). Edaravone is unlikely to be effective in a wider ALS population and there is a sizeable administration burden, limiting excitement for the drug (Hardiman and van den Berg, 2017). Palliative care incorporating dietary and respiratory support, speech and language therapy, and specialist physiotherapy can also improve survival in ALS (Martin et al., 2017b), and provides arguably a greater benefit to quality of life than current pharmaceutical intervention (Hardiman et al., 2011).

In over 20 years since the approval of Riluzole, more than 20 additional compounds have been tested in over 50 randomized, controlled trials, involving in excess of 13,000 ALS patients, with little clinical success (Mitsumoto et al., 2014; Petrov et al., 2017). Consistent with ALS complexity, the tested drugs possess a broad range of proposed mechanisms of action, including anti-inflammation and anti-oxidation. Rather than targeting the underlying genetics, these compounds were trialled for their ability to support the ailing ALS nervous system and restrict the insidious progression of disease. These unsuccessful therapeutics include three neurotrophic factors delivered as recombinant proteins - BDNF, CNTF, and insulin-like growth factor-1 (IGF-1) (Bartus and Johnson, 2017a). Also known as neurotrophins, neurotrophic factors are target-secreted (e.g., from muscles) proteins essential for the growth, development and survival of several nerve types, including motor neurons (Huang and Reichardt, 2001). Delivering neurotrophins has been pursued as a therapy for ALS because their expression can decline with time in models and patients (Krakora et al., 2012). While injection of recombinant proteins is not gene therapy *per se*, when adapted for delivery by viruses, these and other neurotrophic factors have shown promising results in pre-clinical ALS models (Henriques et al., 2010; Nizzardo et al., 2012) (**Table 1**). Additionally, intramuscular injections of plasmids encoding multiple isoforms of hepatocyte growth factor (HGF, drug named VM202) or a transcription factor able to increase expression of vascular endothelial growth factor (VEGF, drug named SB-509) have been trialled in ALS patients (Scarrott et al., 2015). Both plasmids had favorable safety profiles in phase I/II studies (VM202, NCT02039401 and SB-509, NCT00748501) (Sufit et al., 2017), but whether these drugs will be tested further remains unclear (Scarrott et al., 2015).

**Table 1:** Virus-mediated, neuroprotection gene therapies tested in animal models of ALS.

Delivery	Virus-promoter-transgene	Age at injection	Major findings	Animal model	Reference	
**GDNF**						
T/p: MPCs into GC	Rv-PGK1-GDNF (*ex vivo*)	P42	Preserved MN size/number and muscle weight, resulting in improved motor function and extended survival.	SOD1^G93A^ mouse	Mohajeri et al., 1999	


I/M: GC, PS, Q, TA	Ad-CMV-GDNF	P5–7	Preserved MNs, improved motor function, delayed disease onset, and extended survival. No significant benefit in CMAP.	SOD1^G93A^ mouse	Acsadi et al., 2002	


I/M: GC, TB	AAV2-CMV-GDNF	P56	Delayed disease onset, reduced muscle atrophy, preserved MNs, improved motor function, and extended survival.	SOD1^G93A^ mouse	Wang L.-J. et al., 2002	


T/p: hNPCs into lumbar SC	LV-PGK1-GDNF (*ex vivo*)	P100	Robust GDNF expression at end-stage and upregulation of ChAT in the ventral horn, but no significant changes in disease observed.	SOD1^G93A^ rat	Klein et al., 2005	


T/p: hMSCs into DT, TA, TB	LV-PGK1-GDNF (*ex vivo*)	P80 (F)	Control and GDNF hMSCs improved NMJ innervation and preserved MNs. GDNF hMSCs extended survival.	SOD1^G93A^ rat strains	Suzuki et al., 2008	


T/p: MPCs into GC	CMV-GDNF, -VEGF, -IGF-1, and/or -BDNF (*ex vivo*)	P90/P104 /P118	Combined MPC delivery synergistically delayed disease onset, improved motor function and NMJ innervation, and extended survival.	SOD1^G93A^ mouse	Dadon-Nachum et al., 2015	


I/V: tail	AAV9-CAG-GDNF	∼P25	Brain, SC, and limb-muscle GDNF expression. Preserved MNs, increased weight gain and motor function in FL, but not HL.	SOD1^G93A^ rat	Thomsen et al., 2017	


**IGF-1**						


I/M: InC, Q	AAV2-CMV-IFG-1, or -GDNF	P60/P90	IGF-1 delayed disease onset and rate of disease progression, even when delivered symptomatically (P90). GDNF only delayed disease onset.	SOD1^G93A^ mouse	Kaspar et al., 2003	


I/S: lumbar	AAV2-CAG-IGF-1	P60	Preserved MNs and improved motor function, but no difference in microgliosis. Delayed disease onset and extended survival in M.	SOD1^G93A^ mouse	Lepore et al., 2007	


I/C	AAV1-, or AAV2-CMV-IGF-1	P88–90	Preserved MNs, improved motor function, reduced astrogliosis/microgliosis, and extended survival. AAV1 caused better cervical MN preservation, but there was no survival difference between serotypes.	SOD1^G93A^ mouse	Dodge et al., 2008	


I/S: cervical	AAV2-CAG-IGF-1	P80	Preserved MNs. Improved motor function in M. No change in disease onset, disease progression, or survival.	SOD1^G93A^ rat	Franz et al., 2009	


I/CV	AAV4-CMV-IGF-1, and/or -VEGF-165	P80–90	Individually, both improved motor function, and extended survival. No additive effect of combined treatment.	SOD1^G93A^ mouse	Dodge et al., 2010	


I/M: InC, Q	AAV9-CAG-IGF-2	P80	Preserved MNs, improved motor function, induced nerve regeneration, and extended survival.	SOD1^G93A^ mouse	Allodi et al., 2016	


I/M: GC, InC, TA, TB	LV-αCAR-CMV-IGF-1 (MN-specific), or LV-VSV-G-CMV-IGF-1 (muscle-specific)	P28	MN-specific: preserved MNs, improved motor function, delayed disease onset, and extended survival. Muscle-specific: delayed disease onset and improved motor function, but not as well as MN-specific. Gender differences observed.	SOD1^G93A^ mouse	Eleftheriadou et al., 2016	


I/M – Ab, FL, HL, InC, Ma	scAAV9-CMV-IGF-1	P60/P90	Delayed disease onset, preserved MNs, improved motor function, and extended survival.	SOD1^G93A^ mouse	Lin et al., 2016	
**VEGF**						
I/M: D, F, GC, InC, T	LV(EIAV)-VEGF-165, or GDNF	P21/P90	VEGF-165 delayed disease onset, reserved MNs, and extended survival, even when delivered symptomatically (P90). GDNF had little impact on disease phenotypes.	SOD1^G93A^ mouse	Azzouz et al., 2004b	
I/C, I/CV, or I/V: jugular	ssAAV1-PGK1-VEGF, or scAAV9-PGK1-VEGF	P2/P49	I/C resulted in high VEGF expression along entire SC. No VEGF treatment impacted the disease course.	*LIX1^-/-^* cat	Bucher et al., 2013	
T/p: hMSCs into DT, TA, TB	LV-PGK1-VEGF-165, -BDNF, -GDNF, -IGF-1 or -GDNF/-VEGF-165 (*ex vivo*)	P90 (F)	VEGF-165 and GDNF preserved MNs, improved NMJ innervation, and extended survival in isolation, with additive improvements in NMJ innervation and survival when co-delivered.	SOD1^G93A^ rat	Krakora et al., 2013	
I/T	Pseudotyped scAAV9-CMV-VEGF-165	P90	Preserved MNs, improved motor function, reduced microgliosis, and extended survival.	SOD1^G93A^ mouse	Wang et al., 2016	
**Other**						
I/M: DT, GC, TB	Ad-RSV-NT-3 alone or with -CNTF	P3–5	NT3 improved motor function, reduced axonal degeneration, induced muscle re-innervation, and extended survival. CNTF addition provided additive effects.	*pmn* mouse	Haase et al., 1997, 1998	
I/S: lumbar	rAAV-CMV-Bcl-2	P35	Bcl-2 delayed disease onset, preserved MNs, and improved CMAP.	SOD1^G93A^ mouse	Azzouz et al., 2000	
I/M: DT, GC, TB	Ad-RSV-CT-1	P3–5	CT-1 delayed disease onset, weight loss, CMAP decline, and axonal degeneration, improved motor function and muscle weight, and extended survival.	SOD1^G93A^ mouse	Bordet et al., 2001	
I/S: lumbar, or I/M: DT, GC	rAAV1/2-CBA-G-CSF	P70 (F)	I/C delayed disease onset, preserved MNs and NMJs, improved motor function and axon regeneration post-nerve crush, and extended survival. I/M increased plasma G-CSF levels, but failed to transduce MNs.	SOD1^G93A^ mouse	Henriques et al., 2011	
I/M: D, F, HL, InC, T	AAV6-CMV-PRDX3, or -NRF2	P29–31	Neither PRDX3 or NRF2 impacted disease. Poor CNS transduction may have been the cause.	SOD1^G93A^ mouse	Nanou et al., 2013
I/S: lumbar	rAAV2/1-CAG-IL-10	P1	IL-10 did not impact disease onset, but extended survival. Altered immune system genes in CNS at end-stage.	SOD1^G93A^ mouse	Ayers et al., 2015
I/C and I/M: GC	LV-CMV-EEAT2, -GDH2, and/or -NRF2	P65	Individual EAAT2, GDH2, and NRF2 treatments all preserved MNs, but combination therapy also delayed disease onset, improved motor function and body weight, and extended survival.	SOD1^G93A^ mouse	Benkler et al., 2016
I/M: GC	AAV1-CMV-NRG1	P56/P84	NRG1 improved GC but not TA CMAP, preserved NMJs, but not axons or MNs, and increased collateral NMJ sprouting. Had no impact on motor function or disease onset. Effects were reduced when treated later.	SOD1^G93A^ mouse	Mancuso et al., 2016
I/V: tail	AAV9-CMV-DOK7	P90 (M)	DOK7 increased NMJ size and innervation, reduced muscle atrophy, and extended survival, without preserving MNs.	SOD1^G93A^ mouse	Miyoshi et al., 2017
I/T	ssAAV9-CMV-DAO	P90	>2-fold increase in lumbar SC DAO levels. Preserved MNs and axons, reduced microgliosis, and delayed muscle atrophy. Extended survival of F.	SOD1^G93A^ mouse	Wang et al., 2017

*In the *Delivery* column, the mode of delivery (hMSC, human mesenchymal stem cell; hNPC, human neural progenitor cell; I/C, intracerebral; I/CV, intracerbroventricular; I/M, intramuscular; I/S, intraspinal; I/T, intrathecal; I/V, intravenous; MPC, muscle progenitor cell; SC, spinal cord; T/p, transplant) is followed by details of site of injection (Ab, abdominal; D, diaphragm; DT, dorsal trunk; F, facial; FL, forelimb; GC, gastrocnemius; HL, hindlimb; InC, intercostal; Ma, masseter; PS, paraspinal; Q, quadriceps; T, tongue; TA, tibialis anterior; TB, triceps brachii). In the *Virus-promoter-transgene* column, information on the viral vector (Ad, adenovirus; AAV, adeno-associated virus; EIAV, Rabies-G pseudotyped lentiviral-vector; LV, lentivirus; r, recombinant; Rv, retrovirus; sc, self-complementary; ss, single-stranded), gene promoter (CAG, hybrid cytomegalovirus enhancer/chicken β-actin; CBA, chicken β-actin; CMV, cytomegalovirus; PGK1, phosphoglycerate kinase 1; RSV, rous sarcoma virus; VSV-G, Vesicular Stomatitis Virus glycoprotein), and gene of interest (αCAR, coxsackievirus and adenovirus receptor; BDNF, brain-derived neurotrophic factor; Bcl-2, B-cell lymphoma-2; CNTF, ciliary neurotrophic factor; CT-1, cardiotrophin-1; DAO, D-amino acid oxidase; DOK7, docking protein 7; EAAT2, excitatory amino acid transporter 2; G-CSF, granulocyte-colony stimulating factor; GDH2, glutamate-dehydrogenase 2; GDNF, glial-derived neurotrophic factor; IGF-1/2, insulin-like growth factor 1/2; IL-10, interleukin-10; NRF2, nuclear factor erythroid 2-related factor 2; NRG1, Neuroregulin-1; NT-3, neurotrophin-3; PRDX3, peroxiredoxin 3; VEGF, vascular endothelial growth factor) is provided. Acronyms used in additional columns: ChAT, choline acetyltransferase; CMAP, compound muscle action potential; CNS, central nervous system; *LIX*, *limb-expression 1*; F, females only; M, males only; MN, motor neuron; NMJ, neuromuscular junction; P, postnatal day; *pmn*, progressive motor neuronopathy; SOD1, superoxide dismutase 1*.

Without detailed knowledge of disease etiology and underlying cellular pathologies, neuroprotection is potentially the only viable method for tackling a complex syndrome like ALS. However, with increased understanding of gain- and loss-of-function mechanisms of genetic forms of ALS, a second category of knockdown gene therapies encompassing ASOs and RNAi has emerged. These have principally been tested in SOD1, but also C9ORF72, rodent models (**Table 2**). These oligonucleotide-mediated therapeutics are designed to specifically target and reduce levels of toxic, mutant proteins (e.g., C9ORF72, SOD1, TDP-43, FUS) and are showing promise in mice. While they may have a narrow applicability window due to small percentages of genetically determined ALS, given pathological commonalities, such as cytoplasmic TDP-43 sequestration (Neumann et al., 2006) and possible involvement of wild-type SOD1 misfolding in disease (Bosco et al., 2010), there is scope for broader application. Moreover, many sALS patients possess mutations in genes linked to fALS. Targeting the cause of disease in this manner is likely to have the greatest therapeutic impact, and obviates the requirement for co-treatment of multiple downstream pathways. The ASO IONIS-SOD1_Rx_ (a.k.a. ISIS 333611 and BIIB067) targets both wild-type and mutant SOD1 mRNA for degradation (**Figure 2B**). Importantly for this strategy, *Sod1* knockout mice develop normally and do not show motor neuron loss, although their response to axonal injury is impaired (Reaume et al., 1996), and there is evidence that SOD1 loss-of-function may modify ALS severity (Saccon et al., 2013). IONIS-SOD1_Rx_ administration into CSF of SOD1^G93A^ rats resulted in reduced SOD1 protein in spinal cord (Winer et al., 2013). IONIS-SOD1_Rx_ was thus tested in 24 ALS patients in a randomized, placebo-controlled phase I trial (Miller et al., 2013). In this first-in-human clinical study of intrathecal ASO delivery, ≈12 h infusion of IONIS-SOD1_Rx_ was shown to be safe and well tolerated. A phase Ib/IIa trial (NCT02623699) is currently recruiting ALS patients to further evaluate safety, tolerability, and pharmacokinetics of IONIS-SOD1_Rx_.

**Table 2:** Knockdown strategy gene therapies tested in animal models of ALS.

Delivery	Therapeutic	Targeted gene	Treatment age	Major findings	Animal model	Reference
Repeated I/P	ASO	* p75^NTR^*	Starting at P60 (F)	p75^NTR^ levels reduced in lumbar SC, kidneys, and MNs. Delayed disease onset and extended survival, but did not impact disease progression.	SOD1^G93A^ mouse	Turner et al., 2003
Repeated I/P	ASO	*GluR3*	Starting at P50	Delayed disease onset and extended survival, despite lack of GluR3 reduction in the lumbar SC.	SOD1^G93A^ mouse	Rembach et al., 2004
I/M: HL	AAV2-CMV-siRNA	*SOD1*	P45	SOD1 levels reduced in MNs, and motor function improved.	SOD1^G93A^ mouse	Miller et al., 2005
I/M: D, F, HL, InC, T	LV(EIAV)-CMV-shRNA	*SOD1*	P7	SOD1 levels reduced in MNs. Delayed disease onset, preserved MNs, improved motor function, and extended survival.	SOD1^G93A^ mouse	Ralph et al., 2005
I/S: lumbar	LV-PGK1-shRNA	*SOD1*	P40	SOD1 levels reduced in MNs and glial cells. Delayed disease onset, preserved MNs and axons, and improved motor function and CMAP.	SOD1^G93A^ mouse	Raoul et al., 2005
Repeated I/P, or continuous I/CV inf.	ASO	*SOD1*	P65	SOD1 levels reduced in brain and SC by I/CV infusion. Slowed disease progression and extended survival, but did not affect disease onset.	SOD1^G93A^ rat	Smith et al., 2006
Continuous I/T inf.	siRNA	*FasR*	P90	FasR levels reduced in SC. Preserved MNs and axons, improved motor function, and extended survival.	SOD1^G93A^ mouse	Locatelli et al., 2007
I/M: GC, or I/V: tail	rAAV6-H1-shRNA	*SOD1*	P42	I/M delivery targeted MNs and reduced SOD1 mRNA/protein in GC. I/V delivery reduced SOD1 levels in muscle, heart, and liver, and to a lesser extent in SC, but not brain. Disease onset and progression unaffected.	SOD1^G93A^ mouse	Towne et al., 2008
Continuous I/T inf.	siRNA	*SOD1*	≈P85 (M)	SOD1 levels reduced in SC. Delayed disease onset and extended survival.	SOD1^G93A^ mouse	Wang et al., 2008
SN, or I/M: GC	rAd-, or AAV2-U6-shRNA	*SOD1*	P94	Nerve injection more efficient than I/M at MN delivery. rAd reduced SOD1 levels in MNs, slowed disease progression, and extended survival. rAAV2 did not confer any benefit.	SOD1^G93A^ mouse	Wu et al., 2009
I/M: F, FL, HL, InC, T, TC	rAAV6-H1-shRNA	*SOD1*	P1/P5/P15	SOD1 levels reduced in muscles and MNs. Preserved MNs, NMJs, and axon, reduced muscle atrophy, but did not impact neuroinflammation or disease progression.	SOD1^G93A^ mouse	Towne et al., 2011
I/V: tail (P21), or temporal (P1)	AAV9-CAG-shRNA	*SOD1*	P1- 2/P21/P85	P1 injections caused greater reduction than P21 in SOD1 levels in SC, due to more efficient MN transduction. Injections at all ages improved motor function, increased muscle mass, and extended survival, but only P1 delayed disease onset.	SOD1^G93A^ mouse; SOD1^G37R^ mouse	Foust et al., 2013
Repeated I/P	ASO	*AChE*	P35/P84	Treatment at P35 preserved MNs and extended survival, but later delivery had no impact.	SOD1^G93A^ mouse	Gotkine et al., 2013
I/CV: continuous inf.	ASO	*miR-155*	P60	ASO incorporated into brain and SC, and miR-155 target genes impacted. Disease progression slowed and survival extended, but disease onset unaffected.	SOD1^G93A^ mouse	Koval et al., 2013
I/T	AAV2/1-CMV-scFvD3H5	*SOD1*	P45	Sustained expression was observed in MNs. Delayed disease onset, preserved MNs and axons, improved motor function, reduced	SOD1^G93A^ mouse	Patel et al., 2014
I/C	AAV9-H1-shRNA	*SOD1*	P70	SOD1 levels reduced in cortex, but not MNs. Delayed disease onset, preserved MNs and NMJs, improved motor function in hindlimbs, and extended survival.	SOD1^G93A^ rat	Thomsen et al., 2014
I/T	rAAVrh10-CAG-amiR	*SOD1*	P55–60 (F)	SOD1 levels reduced in SC and MNs. Slowed disease progression and extended survival, but disease onset unaffected.	SOD1^G93A^ mouse	Wang H. et al., 2014
I/M: GC (P2), or I/CV (P2), or I/T (P35)	AAV6-CMV-miR, and/or AAV9-gfaABC_1_D-miR, or AAV9-CMV-miR	*SOD1*	P2/P35	I/M (AAV6) delivery reduced SOD1 levels in GC. I/CV delivery resulted in MN (AAV6) and astrocyte (AAV9-gfaABC_1_D) targeting. Both I/CV treatments preserved MNs, and NMJs, improved motor function, decreased muscle atrophy, and extended survival. There was no additive effect. I/T (gfaABC_1_D and CMV) at P35 improved CMAP and motor function, but only CMV preserved MNs and NMJs. Survival was unaffected by I/T.	SOD1^G93A^ mouse	Dirren et al., 2015
I/V: tail	rAAVrh10-U6-miR, or CBA-miR	*SOD1*	P50-68	U6 delayed disease onset and extended survival, while CBA delayed disease progression and extended survival. The effects of U6 were marginally better than CBA. U6 also preserved MNs and improved motor function.	SOD1^G93A^ mouse	Borel et al., 2016
I/CV	ASO	*C90RF7 2*	≈P90/≈P180	Different ASOs decreased repeat-containing C9ORF72 RNA levels in cortex and SC, sense foci in cortex, and poly(GP) and poly(GA) peptides at both time points. Behavioral deficits were alleviated when treated at ≈P180.	*C9ORF72* BAC transgenic mouse (C9^405B^)	Jiang et al., 2016
I/CV (P0), or I/P (P0, P3, and P6), or I/CV and I/V (P85)	MO	*S0D1*	P0/P3/P6/ P85	SOD1 levels reduced in CNS via all routes. I/CV and I/V combination preserved MNs and axons, improved motor function, reduced microgliosis, and extended survival.	SOD1^G93A^ mouse	Nizzardo et al., 2016
I/CV	AAV9-CAG-amiR	*S0D1*	P1	*SOD1* mRNA levels reduced in CNS, muscle, and heart. Preserved MNs, NMJs and axons, delayed SC inflammation, improved motor function and extended survival.	SOD1^G93A^ mouse	Stoica et al., 2016
I/CV	ASO	*Atxn2*	P1	*Atxn2*, but not *TDP-43*, mRNA levels reduced in brain. Improved motor function and extended survival.	*TDP-43^Tg/Tg^* mouse	Becker et al., 2017
I/CV and I/V: temporal (P1), or I/CV and I/V: temporal and/or RbS (P50), or I/S: lumbar (P50)	scAAVrh10-U7-ASO	*S0D1*	P1/P50	All paradigms reduced SOD1 levels in SC. Delayed disease onset and progression, improved motor function, and extended survival at both time points. Neonatal delivery preserved MNs, NMJs, and myofibres, and reduced microgliosis.	SOD1^G93A^ mouse	Biferi et al., 2017
I/V: tail (P21), or temporal (P1)	scAAV9-CAG-shRNA	*S0D1*	P1/P21	Greater MN transduction at P1, shifting to astrocyte tropism at P21, with both reducing SOD1 levels in SC. Both treatments delayed disease onset, improved motor function, and extended survival, but amelioration was better at P1. Genetic suppression of NF-κB in microglia resulted in additive phenotypic improvements.	SOD1^G93A^ mouse	Frakes et al., 2017
I/T	rAAVrh10-CAG-amiR	*S0D1*	P30	Slow and fast injection protocols resulted in different transduction patterns. Both protocols preserved axons, delayed disease onset, and extended survival. Slow injection produced greater phenotypic improvements.	SOD1^G93A^ mouse	Li et al., 2017

*In the *Delivery* column, the mode of delivery (I/C, intracerebral; I/CV, intracerbroventricular; I/M, intramuscular; I/P, intraperitoneal; I/S, intraspinal; I/T, intrathecal; I/V, intravenous; SN, sciatic nerve) is followed by details of site of injection (D, diaphragm; F, facial; FL, forelimb; GC, gastrocnemius; HL, hindlimb; InC, intercostal; inf., infusion; P, postnatal day; RbS, retrobulbar sinus; T, tongue; TC, thoracic cavity). In the *Therapeutic* column, information on the type of therapeutic is provided (Ad, adenovirus; AAV, adeno-associated virus; amiR, artificial microRNA; ASO, antisense oligonucleotide; EIAV, Rabies-G pseudotyped lentiviral-vector; LV, lentivirus; miR, microRNA; MO, morpholino oligonucleotide; r, recombinant; sc, self-complementary; scFvD3H5, a secretable single-chain fragment variable (scFv) antibody composed of heavy and light chain regions of D3H5 monoclonal antibody that binds specifically to misfolded SOD1; shRNA, short hairpin RNA; siRNA, small interfering RNA; ss, single-stranded), along with details on promoter usage (CAG, hybrid cytomegalovirus enhancer/chicken β-actin; CBA, chicken β-actin; CMV, cytomegalovirus; gfaABC_*1*_D, minimal glial fibrillary acidic protein; H1, H1 RNA polymerase III; PGK1, phosphoglycerate kinase 1). Additional acronyms used in other columns include: AChE, acetylcholinesterase; Atxn2, ataxin-2; BAC, bacterial artificial chromosome; CMAP, compound muscle action potential; F, females only; FasR, Fas receptor; GluR3, glutamate receptor subunit 3; M, males only; MN, motor neuron; NMJ, neuromuscular junction; p75^*NTR*^, p75-neurotrophin receptor; SC, spinal cord; SOD1, superoxide dismutase 1; Tg, transgene*.

## SMA Gene Therapy Lessons for ALS

There are numerous possible intersecting explanations for the plethora of failed ALS clinical trials (Mitsumoto et al., 2014), not limited to (1) most pre-clinical research has been conducted in SOD1^G93A^ mice, which do not accurately model the entire ALS spectrum; (2) poor experimental design and execution of pre-clinical work (Scott et al., 2008); (3) focusing on mouse survival as an indicator of drug potential (Genç and Özdinler, 2014); (4) pharmacological issues such as insufficient dose or access/bioavailability to targeted tissue; (5) timing of intervention (Benatar, 2007); (6) patient heterogeneity, poor trial stratification, and scarcity of biomarkers; and (7) incomplete understanding of disease mechanism(s).

Similar difficulties have, at least partially, been overcome by the SMA research community in order for nusinersen to receive regulatory approval. Being a monogenic condition, treating SMA is undoubtedly simpler than the challenge posed by the broad-ranging heterogeneity of ALS. Nonetheless, the clinical approval of nusinersen was a significant milestone not just for SMA, but gene therapy as a whole. That is not to say that the job is complete for SMA, as the clinical response to nusinersen is wide-ranging and includes non-responders (Finkel et al., 2017). However, over the last decade, a great deal has been learnt from the pre-clinical development of SMA gene therapies and the clinical trials of nusinersen and AVXS-101. Given the clinical and mechanistic overlap between the diseases, these lessons learned from SMA may be useful for ALS, particularly when considering the array of gene therapies pre-clinically tested in ALS rodent models (**Tables 1**, **2**) and thus likely to be in the clinical drug pipeline. We have therefore summarised this information to emphasize some key points for ALS gene therapy development.

### Careful Therapeutic Targeting Is Required

A clear understanding of where a therapy is needed is mandatory for clinical success. SMA is primarily a LMN disorder; however, in severe cases, pervasive pathology has been reported. For instance, congenital heart problems (Rudnik-Schöneborn et al., 2008), bone complications (Khatri et al., 2008), and vascular defects (Somers et al., 2016) are known to occur in some type I SMA patients. Cell intrinsic SMN depletion causes similar and additional pathologies in mice (Hamilton and Gillingwater, 2013). A sliding scale of vulnerability to SMN reduction has therefore been suggested; at one end, LMNs are the first cell type disturbed by diminished SMN, and as levels are decreased further, more cell types and tissues become affected (Sleigh et al., 2011). This appears to be the case in mouse models, but may be sub-clinical in the majority of SMA patients, as non-motor neuronal involvement is less common. Nevertheless, concerns persist that treating SMA patients with nusinersen, which is delivered directly to the CSF to target LMNs, may alter disease trajectory and reveal novel pathologies caused by chronic SMN deficiency outside the CNS (Tizzano and Finkel, 2017).

There is thus discussion, for both ASOs and viruses, as to which single delivery route provides the best therapeutic outcome for SMA (Hua et al., 2011; Glascock et al., 2012b; Porensky et al., 2012; Nizzardo et al., 2014; Zhou et al., 2015). Major determinants of this are the BBB and the BSCB, which not only restrict access of systemically delivered therapies to the brain and spinal cord, but can also confine drugs within the CNS when directly administered. Targeting therapies to NMJs for uptake and retrograde transport along motor axons can circumvent these barriers (Tosolini et al., 2013; Mohan et al., 2014; Tosolini and Morris, 2016), but this is compromised by denervation. Nonetheless, once inside the CNS, ASOs are able to distribute widely (Rigo et al., 2014). Accordingly, intracerebroventricular injection of SMN ASOs results in robust SMN upregulation in the CNS (Hua et al., 2010; Passini et al., 2011; Porensky et al., 2012; Rigo et al., 2014), while intravenous or subcutaneous administration triggers a more systemic increase outside the CNS (Hua et al., 2011; Keil et al., 2014). Viral vectors are better than ASOs at overcoming BBB permeability problems (Foust et al., 2010; Valori et al., 2010; Dominguez et al., 2011; Meyer et al., 2015), but distinctions in therapeutic outcome between routes still apply (Glascock et al., 2012a,b). In addition, modifying the delivery speed of intrathecal injections can alter AAV tropism; slower injections (i.e., over 8 min) result in preferential transduction of the spinal cord, whereas faster injections (i.e., over 30 s) preferably transduce the brain and peripheral tissue (Li et al., 2017). Notwithstanding, there remains little doubt that using multiple injection modes to provide body-wide SMN augmentation provides the best phenotypic rescue in SMA mice (Nizzardo et al., 2014; Osman et al., 2014), while limiting the onset of delayed, non-neuronal pathologies such as tail necrosis (Foust et al., 2010; Dominguez et al., 2011). The importance of careful therapeutic targeting of SMN to all required cells has been confirmed using numerous transgenic mice where SMN has been overexpressed in SMA models in a tissue-specific fashion (Gogliotti et al., 2012; Martinez et al., 2012).

Cautious selection of administration route is not the only way to affect the voyage of gene therapy through the body. A number of additional therapy-dependent tactics can be employed to optimize delivery. Despite limited packaging capacity (≈4.5 kb for single-stranded and ≈2.4 kb for self-complementary AAV), AAV has become the most promising vector for gene delivery in neurological disease; it establishes stable nuclear episomes, thus reducing the risk of integrating into the host genome and causing insertional mutagenesis, it can transduce both dividing and non-mitotic cells, and it maintains exogenous gene expression for extended periods (Murlidharan et al., 2014). With approximately twice the capacity of AAV, LV has also been employed as a proof-of-concept vector in pre-clinical models of SMA (Azzouz et al., 2004a) and ALS (**Tables 1**, **2**); however, given that LV can randomly insert into the host genome, there are major safety issues associated with its clinical application (Imbert et al., 2017). The advantages of AAV led to scAAV9 being chosen for *SMN1* delivery in the AveXis gene therapy, AVXS-101. AAV serotypes possess divergent capsid proteins that bind to distinct host cell surface receptors and co-receptors, thereby determining the cells a virus can transduce (i.e., the tropism) and how efficiently it can spread (Murlidharan et al., 2014). Multiple AAV serotypes have been used in SMA mice (Foust et al., 2010; Passini et al., 2010; Tsai et al., 2012), but serotype 9 was selected for AVXS-101 because of its comparatively strong tropism toward LMNs throughout the spinal cord in a range of species (Foust et al., 2009; Bevan et al., 2011; Federici et al., 2012). While numerous AAV serotypes and administration routes have also been tested in ALS mice (**Tables 1**, **2**), it remains unclear which serotype will prove to be most effective. AAV9 and AAVrh10 are good candidates, but natural and engineered serotypes have recently been identified with improved tropism toward ALS-pertinent tissues (Deverman et al., 2016; Chan et al., 2017). Viral transgene expression can also be restricted using promoters with selective and defined expression patterns (Kügler, 2016). Combining knowledge of viral tropism with promoter selectivity thus provides a potential method for exquisite, cell type-specific targeting (von Jonquieres et al., 2013). Disease state (Chen et al., 2012) and age (Foust et al., 2009; Tosolini and Morris, 2016) also impact viral tropism, likely through alterations in host cell receptor availability, while promoter usage likewise changes with pathology and time; hence, it will be vital to test permutations of virus serotype/promoter in relevant pre-clinical models of ALS throughout all stages of disease.

Unlike AAV, ASOs do not readily penetrate tissues, while their cellular uptake and transition to the nucleus, their site of action, is limited. Indeed, it is estimated that <1% of ASOs reach their desired target, as the majority distribute to unwanted organs such as the liver (Godfrey et al., 2017). This mandates repeated therapeutic injections that can cause adverse events (Haché et al., 2016) and toxic ASO accumulation (Godfrey et al., 2017). Consequently, several chemical modifications to the ASO phosphate backbone have been tested and shown to improve safety and pharmacological properties (Evers et al., 2015). Moreover, a number of drug distribution systems compatible with systemic delivery are being developed that enhance ASO transport to disease-pertinent tissues and therefore ease administration and reduce the required dose. These ASO vectorisation strategies can be divided into (1) viral approaches that use harmless, non-replicating viruses, such as AAV, and (2) non-viral strategies that utilise different positively charged molecules such as lipids or peptides (Lehto et al., 2012). Viruses present immunogenicity issues and are not suitable for all nucleic acid-based molecules; nevertheless, to aid cellular targeting and cytoplasm-to-nucleus transport, viral vectors have been engineered to encode modified snRNAs that incorporate specific ASO sequences. Once inside target cells, these ASOs are imported into the nucleus where they accumulate as part of snRNPs (Imbert et al., 2017). When packaged into viral vectors, snRNAs containing ASOs targeting *SMN2* have shown potential for SMA (Meyer et al., 2009; Dal Mas et al., 2015; Odermatt et al., 2016). As has the non-viral approach of conjugating ASOs to CPPs (Hammond et al., 2016; Shabanpoor et al., 2017). CPPs are 5–30 amino acid long, positively charged peptides that transport various macromolecules across cell membranes (Lehto et al., 2012). Highlighting the significance of therapeutic targeting, intravenous injection of an *SMN2* SSO conjugated to a CNS-targeting CPP caused the greatest extension in SMA mouse survival reported to date – from 12 to 456 days compared to only 54 days for the “naked” oligonucleotide (Hammond et al., 2016). Importantly, these CPP-oligonucleotides are capable of delivery to the CNS of both neonatal and adult mice (Hammond et al., 2016; Shabanpoor et al., 2017); however, the safety, tolerability, and pharmacokinetics of CPP-conjugated ASOs are yet to be tested in additional organisms.

AAV and LV have separately been combined with RNAi-based strategies for SOD1 knockdown (**Table 2**), but only recently have ASO vectorisation strategies been tried in ALS models. Compared with non-encapsulated oligonucleotides, loading *SOD1*-specific ASOs into lipid particles caused a much greater reduction in SOD1 protein in HEK293 cells (Chen et al., 2017). Moreover, direct intravascular delivery of non-ASO-loaded nanoparticles resulted in brain accumulation in wild-type zebrafish, indicating promise for future work in ALS mice (Chen et al., 2017). *SOD1* pre-mRNA-targeting ASOs were also embedded in modified snRNAs and engineered into AAVrh10, which was then co-injected into the blood and brain of SOD1^G93A^ mice either at birth or pre-symptomatically at 50 days (Biferi et al., 2017). The ASO skips *SOD1* exon 2 generating a premature stop codon, which resulted in ≈70% reduction of SOD1 protein levels in the spinal cord 112 days post-perinatal administration. The gene therapy caused vast improvements in neuromuscular function, restricted weight loss, and, when given early, resulted in the greatest SOD1^G93A^ survival extension yet reported to ≈250 days (Biferi et al., 2017).

Amyotrophic lateral sclerosis patients and models do not show the same cell and tissue vulnerability-resistance continuum as SMA; however, it is clear that pathology is not limited to LMNs and UMNs, probably contributing to the gamut of unsuccessful ALS clinical trials (Bartus and Johnson, 2017a). Indeed, the ALS/FTD clinicopathological overlap (Morita et al., 2006; Neumann et al., 2006; Vance et al., 2006) has been replicated in ALS mice, indicating the importance of cortical cells and synapses to the disease (Fogarty et al., 2015, 2016). Furthermore, the non-cell autonomous toxicities emanating from cells such as microglia (Puentes et al., 2016), and the prion-like cell-to-cell spread of pathological aggregates, also indicate that targeting therapies to motor neurons as well as additional cells and tissues is likely to be required in order to generate meaningful improvements in ALS prognosis. Indeed, disease onset and mortality are delayed in mutant SOD1 mice when microglial activation is pharmacologically restricted prior to disease onset (Kriz et al., 2002; van den Bosch et al., 2002), while disease progression is slowed in mutant SOD1 mice in which SOD1 is removed from microglia, oligodendrocytes, or astrocytes (Boillée et al., 2006; Yamanaka et al., 2008; Kang et al., 2013). Moreover, expression of mutant SOD1 in motor neurons alone is insufficient to fully recapitulate the mutant SOD1 mouse phenotype (Lino et al., 2002; Pramatarova et al., 2001), while deletion of *SOD1* from motor neurons and interneurons of SOD1 mutant mice only delays disease onset (Wang et al., 2009).

Spinal muscular atrophy research has highlighted the importance of comparing different therapy injection sites and testing novel technologies to improve targeting of therapeutics not only to the required tissues, but also to the correct resident cells and subcellular locations. Viral targeting of rapidly dividing cells affected by ALS (e.g., astrocytes), subtleties in motor neuron subtype vulnerability (Nijssen et al., 2017), along with the indication that different subcellular motor neuron compartments, such as the NMJ, may require differential support and treatment (Moloney et al., 2014), add to the complexity of the challenge ahead. It is therefore imperative that during ALS therapy design, careful consideration is given to what cells and tissues need to be targeted and how exactly this can be most effectively achieved.

### Combinatorial Treatment Is Key

It is conceivable that carefully crafted gene therapy combinations targeting multiple disease mechanisms could provide additive effects in MNDs. Indeed, AAV1-follistatin treatment significantly boosted muscle and body weight of SMA mice suboptimally dosed with *SMN2* ASO (Feng et al., 2016), while in isolation, follistatin upregulation or myostatin reduction have little effect (Sumner et al., 2009; Rindt et al., 2012). Co-delivery of recombinant follistatin with an *SMN2*-inducing compound also resulted in a small additive enhancement in motor function, but not survival (Harris and Butchbach, 2015). Similarly, genetic upregulation of SMA modifier plastin 3 has no effect on untreated SMA mouse lifespan (Ackermann et al., 2013), but can drastically improve survival in ASO-dosed animals (Hosseinibarkooie et al., 2016). A range of other pathological mechanisms could be concomitantly targeted in SMA (Bowerman et al., 2017). Combinations of drugs directed at just the SMN pathway have also proven more effective than individual treatments (Kwon et al., 2011; Liu et al., 2013), while the impact of ASOs targeting *SMN2* has been enhanced by co-treatment with a compound (Osman et al., 2017), co-targeting the *SMN2*-repressing long non-coding RNA, *SMN-AS1*, for ASO-mediated knockdown (d’Ydewalle et al., 2017; Woo et al., 2017), and co-masking additional negative *SMN2* splicing elements (Pao et al., 2013). Furthermore, a holistic approach to treatment appears to be important, as providing nutritional support to SMA mice can advance therapeutic efficacy (Narver et al., 2008; Butchbach et al., 2014).

These SMA studies suggest that previously unsuccessful therapeutics may provide additive benefits when used in combination, highlighting the importance of simultaneously treating multiple disease pathways. Given the pathology observed in ALS, it will also be vital to co-modify pathways in different cell types. To facilitate this, viral vectors engineered with selective promoters could be united with CPP-conjugated ASOs in order to synergistically target multiple genes in related and independent pathways across distinct and highly specific cell populations. This was recently confirmed in mutant SOD1^G93A^ mice by concomitantly targeting distinct disease mechanisms in motor neurons, astrocytes and microglia (Frakes et al., 2017). In isolation, genetic suppression of the NF-κB pathway in microglia or shRNA-mediated knockdown of *SOD1* in motor neurons and astrocytes via systemic AAV9 administration resulted in similar improvements in survival, disease onset, and progression of mutant SOD1 mice. However, the combined targeting of these two pathomechanisms across three major cell types resulted in an additive amelioration in all assessed phenotypes. The median mutant lifespan was expanded from 137 to 188 days with a maximum survival of 204 days, which is one of the best extensions reported to date (Frakes et al., 2017). In a separate study, joint lentiviral targeting of three distinct disease pathways aiming to reduce excitotoxicity resulted in a synergistic neuroprotective effect in SOD1^G93A^ mice (Benkler et al., 2016), while simultaneous delivery of multiple neurotrophic factors can also induce modest additive effects over individual therapies (Haase et al., 1997; Krakora et al., 2013; Dadon-Nachum et al., 2015), although not consistently (Dodge et al., 2010). Like SMA, it is thus clear that prognosis can be improved in ALS models by attempting a multifaceted gene therapy approach. It is somewhat surprising that there have not yet been any published studies that test concurrent delivery of neuroprotective and knockdown strategies *in vivo* for ALS. ASO-mediated depletion of toxic mutant proteins is likely to be most critical in ALS, and should be joined with a gamut of neuroprotective accessory therapies such as neurotrophic factors (Henriques et al., 2010), SMN upregulation (e.g., via nusinersen or AVXS-101) (Kariya et al., 2012; Turner et al., 2014), and preservation of NMJ innervation (Miyoshi et al., 2017), in order to determine the most efficacious drug combinations.

### Consider Dose Number and Drug Concentrations

In addition to delivering gene therapies to the intended cellular and subcellular site(s), effectors must be expressed/released at a therapeutically viable concentration. Providing multiple *SMN2* ASO doses results in greater phenotypic amelioration in SMA mice (Hua et al., 2011; Zhou et al., 2015; Hammond et al., 2016), while increased concentrations of ASOs or AAVs have a similar positive effect (Meyer et al., 2015; Hammond et al., 2016; Hosseinibarkooie et al., 2016). These results unsurprisingly indicate that delivering greater quantities of therapy, results in higher SMN upregulation, and therefore a better phenotypic rescue of SMA mice. There will inevitably be a point at which SMN overexpression becomes detrimental to cells; however, two-fold genetic overexpression of *SMN* in the nervous system of control mice appears to be safe (Turner et al., 2014; Perera et al., 2016). Nevertheless, this may not be the case for all genes (Denovan-Wright et al., 2008), and thus care must be taken when artificially increasing protein abundance, especially considering that secreted proteins can elicit autocrine and paracrine effects (Baumgartner and Shine, 1997).

Caution must also be exercised with target gene reduction strategies as there may be disparate, time-dependent consequences between protein reduction below a physiological threshold and complete absence (Rossi et al., 2015). That being said, toxicities associated with ASO or AAV accumulation are likely to arise before viability is perturbed by excessive modulation of a gene. Hence, there is a fine balance between administering sufficient gene therapy to ensure correct targeting in effective quantities without causing systemic toxic accumulation and adverse side effects. Upon intravenous injection, ASOs accumulate in the liver, kidneys, and lymph nodes, amongst other places, and can cause hybridisation-independent toxicities (Godfrey et al., 2017), while AAVs can become similarly enriched (Zincarelli et al., 2008). The methods to enhance delivery to disease-susceptible cells and tissues discussed above (e.g., ASO-CPP conjugation) will undoubtedly aid in this battle, and should be optimized in ALS mice, along with ASO chemistries, to enrich the appropriately targeted therapeutic load. Moreover, multiple ASO doses and increasing concentrations of gene therapies must be tested in relevant models to identify the most effective and safe therapeutic regimes. Due to the immune response, repeated AAV dosing is not practical (although immunomodulation is an option) (Lorain et al., 2008), but ASOs can be administered at multiple time points. However, ASO concentration must also be optimized to escape host immune responses (Wang et al., 2008). It should also be remembered that once an AAV has been delivered, relatively little can be done to regulate transgene expression, but ASOs can be neutralized by sequestration using complimentary decoy ASOs (Rigo et al., 2014; Hua et al., 2015).

### Therapeutic Timing Is Critical

As disease progresses, the number of impacted proteins and processes will likely increase as a result of time as pathways diverge from the initial cause of pathology. This provides a rationale for why selectively targeting individual disease pathways toward the end of a cascade may provide only limited benefit. Moreover, the greater the duration over which a disease develops, the more anatomical, circuit, and cellular damage will ensue leading to greater loss-of-function and thus a more significant challenge of recovery (Ramírez-Jarquín et al., 2014; Bartus and Johnson, 2017b). For example, early NMJ denervation followed by LMN die-back will severely, if not totally, restrict the ability of the LMN to respond to extracellular neurotrophic signaling. The successful treatment of any disorder is thus more likely to occur when a therapy is administered during early pathogenesis rather than at later time points and, in particular, at disease end stage. Whilst intuitive, this highlights the importance of earlier diagnosis, especially for ALS.

Indeed, the earlier SMN levels are augmented in SMA mice via *SMN* gene therapy, the better the therapeutic outcome (Foust et al., 2010; Hua et al., 2010, 2011; Porensky et al., 2012; Bogdanik et al., 2015; Zhou et al., 2015). Age-dependent differences in BBB permeability, neuropil density, and cell tropisms may contribute (Foust et al., 2009, 2010); however, the early temporal requirement for SMN has been corroborated using mice with inducible *SMN* alleles (Le et al., 2011; Lutz et al., 2011; Kariya et al., 2014a), and is consistent with human and mouse *SMN* being most highly expressed in the CNS perinatally (Jablonka and Sendtner, 2017), and the most common and severe form of SMA (type I) manifesting before 6 months of age. Accordingly, artificially reducing SMN levels in young adult mice has fewer repercussions than when SMN is diminished at earlier time points (Le et al., 2011; Kariya et al., 2014a). All of this suggests that there is a therapeutic window of opportunity during early development in which *SMN* gene therapy is likely to have the greatest chance of success. One study indicates that in mice, the postnatal window of highest SMN requirement coincides with neuromuscular maturation, and that NMJ disruption causes SMN upregulation in motor neurons (Kariya et al., 2014a). Accordingly, nusinersen is being tested in the open-label NURTURE trial in pre-symptomatic newborns genetically diagnosed with SMA. Interim analyses appear promising, and when complete are likely to provide compelling evidence of the importance of treating SMA as early as possible. It is perhaps for this reason, that a number of small molecule drugs that have proven useful in SMA mice dosed from birth, have failed in clinical trials in which patients are treated post-symptom onset (Fuller et al., 2010). Nevertheless, post-symptomatic restoration of SMN using an inducible allele has been shown to reverse overt neuromuscular pathology and significantly improve SMA mouse lifespan (Lutz et al., 2011). This was corroborated using systemic administration of *SMN2*-targeting ASOs in a mild SMA mouse model (Bogdanik et al., 2015), and in SMA mice sub-optimally dosed with a small molecule *SMN2* splice-modifying drug and subsequently re-treated with the same compound or AAV1-follistatin gene therapy (Feng et al., 2016).

Early therapeutic interventions in mutant SOD1 mice also often result in greater impact on disease. For example, pre-symptomatic injection of VEGF-expressing LV into SOD1^G93A^ mouse muscles resulted in greater delay in disease onset and progression compared to injection at paralysis onset (Azzouz et al., 2004b). The same is true for AAV-mediated IGF-1 delivery (Kaspar et al., 2003) and SOD1 silencing (Foust et al., 2013; Biferi et al., 2017), and recombinant VEGF injections into the brain of mutant SOD1 rats (Storkebaum et al., 2005). Significantly, these studies show, like in SMA, that survival of mutant SOD1 rodents can be extended even when therapies are delivered post-symptomatically. There is also evidence to indicate that treatment during the perinatal stage (e.g., postnatal day 1, P1) can cause even greater improvements in SOD1^G93A^ lifespan (Foust et al., 2013; Biferi et al., 2017), perhaps due in part to sub-clinical embryonic/perinatal defects (van Zundert et al., 2012) and differential age-dependent gene therapy tropism (Foust et al., 2009); however, testing intervention timing in these models needs to be therapeutically appropriate, as ALS is an adult-onset neurodegenerative disease currently with no biomarkers. The pre-symptomatic treatment of fALS patients with a known ALS-causing genetic mutation is a possibility, in the same vein as SMA patients in the NURTURE trial of nusinersen, but treatment in humans at an age akin to P1 is highly unlikely. Nevertheless, perinatal therapy administration paradigms provide useful proof-of-concept information, and are invaluable in gene therapy optimisation.

Amyotrophic lateral sclerosis greatly impacts the neuro-muscular system and its proteome, which could cause disease-specific, time-constrained alterations that affect therapeutic efficacy; for instance, viral tropism could be impacted through differential receptor expression (Tosolini and Morris, 2016). Rodent work indicates that there is likely to be an optimal period for ALS therapy delivery, but with a broader therapeutic window, in which disease progression can at least be slowed, if not halted or even partially reversed. It is therefore paramount that potential therapeutics are tested at a range of time points in ALS rodents and in large animals including non-human primates in order to improve therapeutic timing strategy.

## Conclusion

Typified by extensive heterogeneity, the ALS disease spectrum poses a daunting challenge for developing effective treatments. This diversity, along with numerous other factors, has resulted in an overabundance of unsuccessful clinical trials. Many of these involved compounds targeting likely secondary pathogenic pathways with only limited therapeutic potential. However, over the last two decades, gene therapy using ASOs or viral vectors have emerged as the most promising strategy for treating nervous system disorders. The predominantly LMN disease SMA has benefited from this burgeoning field, with the recent regulatory approval of the ASO nusinersen. Through developing ASO- and virus-mediated drugs for SMA, much has been learnt about gene therapy design and development that could help to alleviate the impact of other MNDs. Pre-clinical SMA research has made it clear that gene therapies must be efficiently delivered to pertinent sites of pathology, at concentrations within the therapeutic range, and at appropriate times in order to increase the chances of success. Tantamount to this is the parallel modification of multiple disease pathways across cell and tissue types. Similar conclusions are also beginning to emerge from pre-clinical ALS models. It is thus by no coincidence that the greatest amelioration of the SOD1^G93A^ mouse phenotype to date was driven by the combined targeting of two pathomechanisms across multiple cell types, and the dual-administration of an AAV-guided *SOD1*-specific ASO into the blood and brain. Given the current lack of diagnostic and prognostic biomarkers for ALS and reliance upon the SOD1^G93A^ mouse, successful translation to patients will be tricky. Nevertheless, by considering issues outlined in this review and thinking clearly about treatment logistics, a viable ALS gene therapy is unlikely to be far from the clinic.

## Author Contributions

APT and JNS wrote the manuscript, and have approved submission of this work.

## Conflict of Interest Statement

The authors declare that the research was conducted in the absence of any commercial or financial relationships that could be construed as a potential conflict of interest.

## Abbreviations

AAV: adeno-associated virus
ALS: amyotrophic lateral sclerosis
ASO: antisense oligonucleotide
BBB: blood-brain barrier
BDNF: brain-derived neurotrophic factor
BSCB: blood-spinal cord barrier
C9ORF72: chromosome 9 open reading frame 72
CNS: central nervous system
CNTF: ciliary-derived neurotrophic factor
CPP: cell-penetrating peptide
CSF: cerebrospinal fluid
EMA: European Medicines Agency
fALS: familial ALS
FDA: Food and Drug Administration
FTD: frontotemporal dementia
FUS: fused in sarcoma
HGF: hepatocyte growth factor
IGF-1: insulin-like growth factor
iPSC: induced pluripotent stem cell
ISS-N1: intronic splicing silencer N1
LMN: lower motor neuron
LV: lentivirus
MND: motor neuron disease
NMJ: neuromuscular junction
RNAi: RNA interference
sALS: sporadic ALS
scAAV: self-complementary AAV
SMA: spinal muscular atrophy
SMN: survival motor neuron
snRNA: small nuclear RNA
snRNP: small nuclear ribonucleoprotein
SOD1: superoxide dismutase 1
SSO: splice-switching oligonucleotide
TARDBP: transactive-region DNA binding protein
TLS: translocated in liposarcoma
UMN: upper motor neuron
VEGF: vascular endothelial growth factor.
